# Epidemiology and resistance mechanisms of tigecycline- and carbapenem-resistant *Enterobacteriaceae* in China: a multicentre genome-based study

**DOI:** 10.3389/fmicb.2025.1582851

**Published:** 2025-05-07

**Authors:** Lixin Yan, Tingting Ma, Wen Wang, Zhen Cai, Hong Du, Zhongju Chen, Renru Han, Yan Guo, Gang Li, Wei Jia, Jia Tao

**Affiliations:** ^1^The First Clinical Medical College of Ningxia Medical University, Yinchuan, China; ^2^Laboratory Medical Center, General Hospital of Ningxia Medical University, Yinchuan, China; ^3^Ningxia Key Laboratory of Clinical and Pathogenic Microbiology, Yinchuan, China; ^4^Department of Clinical Laboratory, Aviation General Hospital, Beijing, China; ^5^Department of Clinical Laboratory, The Second Affiliated Hospital of Soochow University, Suzhou, China; ^6^Department of Laboratory Medicine, Tongji Hospital, Tongji Medical College, Huazhong University of Science and Technology, Wuhan, Hubei, China; ^7^Institute of Antibiotics, Huashan Hospital, Fudan University, Shanghai, China; ^8^Key Laboratory of Clinical Pharmacology of Antibiotics, Ministry of Health, Shanghai, China

**Keywords:** CRE, tigecycline resistance, genemic analysis, *tmexCD2-toprJ2*, *tet*(A)

## Abstract

**Objectives:**

To elucidate the molecular epidemiology of tigecycline and carbapenem-resistant *Enterobacteriaceae* isolates and mechanisms of tigecycline resistance.

**Methods:**

We gathered 31 unduplicated strains of tigecycline-resistant *Enterobacteriaceae* from six hospitals nationwide. Antimicrobial susceptibility testing, phenotypic detection, and PCR identification were performed first, followed by homology analysis using MLST and PFGE. Conjugation transfer experiments using resistance gene plasmids were carried out, and the conjugates’ growth curves were examined. All strains were sequenced using the Illumina HiSeq technology, and we identified a strain KP28 carrying a complete gene cluster *tmexCD2-toprJ2*. Then, its plasmid was further constructed using the PacBio platforms to complete the frame. The genetic connection of the *tmexCD2-toprJ2* gene cluster carried by KP28 was established using core genome analyses.

**Results:**

All 31 tigecycline-resistant *Enterobacteriaceae* strains (TG-CRE) were multidrug resistant. PFGE classified strains of CRKP, CRECL, and CRKAE into 16 distinct spectra, 6 distinct spectra, and 3 distinct spectra. MLST results showed a high concentration of ST11 in CRKP strains and a predominance of ST116 in CRECL strains, suggesting possible clonal transmission or selective dominance. The findings of the plasmid conjugation assay revealed that three strains expressing carbapenem resistance genes were effectively transmitted to the recipient cell *E. coli* EC600. WGS data revealed that these 31 strains include 79 resistance genes, with one strain (KP28) carrying the whole tigecycline resistance gene cluster, *tmexC2D2-toprJ2*. This resistance gene is contained in a large IncHI5 plasmid, which is difficult to transfer.

**Conclusion:**

The overall carriage rate of the *tmexC2D2-toprJ2* gene cluster was found to be low among the five Chinese hospitals investigated. Conversely, *tet*(A) mutations were present in most of the strains. Bacteria with the carbapenem resistance genes *bla*_KPC_ and *bla*_NDM_ are vulnerable to horizontal transmission. Increasing the risk of transmission of antibiotic-resistant genes.

## 1 Introduction

With a high global morbidity and mortality rate and few available treatments, carbapenem-resistant *Enterobacteriaceae* (CRE) infections have emerged as a major public health concern (van Duin, 2017). The third-generation antibiotic in the tetracycline family, tigecycline, has a wider spectrum of action against both Gram-positive and Gram-negative bacteria that are extensively drug-resistant (XDR) (Chopra and Roberts, 2001). For the treatment of CRE infection, tigecycline is now considered a last-resort antibiotic. According to an international ESCMID cross-sectional survey, the most popular treatment for patients with CRE-caused skin and soft-tissue infections, as well as intra-abdominal infections, is tigecycline monotherapy (Papst et al., 2018). Brust et al. (2014) reported a favorable outcome in the treatment of CRE urinary tract infection with high dose tigecycline. However, recently reported studies have revealed the emergence of tigecycline resistance in CRE strains from humans and animals worldwide (Chiu et al., 2017; Cui et al., 2021; Hirabayashi et al., 2021). Tigecycline resistance has become a serious concern and poses a major threat to public health.

Tigecycline reversibly binds to the 30S subunit of bacterial ribosomes, inhibiting the extension of the peptide chain, thus preventing bacterial protein translation (Yaghoubi et al., 2022). To date, several mechanisms associated with resistance to tigecycline have been reported (Korczak et al., 2024). The most commonly reported mechanism in Gram-negative bacteria is the increased expression of efflux pump families. Resistance-nodulation-cell division (RND) efflux pump is the most important molecular mechanism in the resistance to tigecycline, such as AcrAB, *tmexCD1-toprJ1*, *tmexCD2-toprJ2* and OqxAB (Yaghoubi et al., 2022). The expression of the AcrAB efflux pump is controlled by several transcriptional activators of the AraC/XylS family, such as RamA, MarA, RarA, and SoxS, which can increase the production of AcrAB proteins and enhancing efflux by interacting with the acrAB promoter (Ruzin et al., 2008; Veleba et al., 2012). These transcriptional activators are regulated by themselves as trans-acting repressors. Overproduction of another RND efflux system, OqxAB, is also regulated by the local repressor OqxR (De Majumdar et al., 2013). Mobile gene cluster *tmexCD-toprJ* is a kind of plasmid-located RND efflux pump genes and has been found to be carried by different plasmid types. *TmexCD-toprJ* complexes function as efflux pumps for tigecycline that could confer resistance to tigecycline (Liu et al., 2022). In addition, mutations of other genes, such as *tet*(A), *rpsJ*, *adeS*, *rpoB*, and *rrf*, have been reported to be associated with decreased tigecycline susceptibility (Hua et al., 2021; Xu et al., 2021; He et al., 2018).

Recently, tigecycline resistance with multiple mechanisms has raised a major concern worldwide. This study aimed to investigate the phenotypic characteristics, molecular epidemiology, and mechanisms of tigecycline resistance in CRE isolates from six hospitals in China.

## 2 Materials and methods

### 2.1 Bacterial isolate collection and identification

A total of 31 unduplicated tigecycline-and carbapenem-resistant *Enterobacteriaceae* (CRE) strains were collected from six hospitals across Beijing, Shanghai, Wuhan, Suzhou, Chengdu, and Yinchuan in China between January and April 2023. All of the samples and clinical isolates were generated as part of routine hospital laboratory procedures. The distribution of specimen types was mostly sputum (48.3%, 14/29), followed by urine (24.1%, 7/29), pus (17.2%, 5/29), blood (6.9%, 2/29), and cerebrospinal fluid (3.5%, 1/29). All strains were identified using MALDI-TOF-MS (bioMérieux, France), confirming that all bacteria included in the study were members of the *Enterobacteriaceae* family. The distribution of bacterial strains was dominated by *Klebsiella pneumoniae* (64.52%, 20/31), followed by *Enterobacter cloacae* (25.80%, 8/31) and *Enterobacter aerogenes* (9.68%, 3/31). To identify carbapenemase production in all CRE strains, the enzyme inhibitor-enhanced assay (PBA-EDTA method) was employed (Tsakris et al., 2010). PCR amplification was utilized to detect four common carbapenemase-encoding genes: *bla*_KPC_, *bla*_IMP_, *bla*_NDM_, and *bla*_OXA-48_ (Supplementary Table S1), followed by Sanger sequencing and analyzed using BLAST^1^ (Tian et al., 2023). Approval by Institutional Review Board or Institutional Animal Care and Use Committee was not required because our research was not involved with human subjects or animal experimentation.

### 2.2 Antimicrobial susceptibility testing (AST)

According to the Clinical and Laboratory Standards Institute (CLSI) guidelines, an antimicrobial susceptibility test was performed using the VITEK® 2 Compact to determine MICs of piperacillin/tazobactam, ceftazidime, cefepime, aztreonam, imipenem, meropenem, amikacin, ciprofloxacin, levofloxacin. All antimicrobials were interpreted according to the standard of the CLSI document (Clinical and Laboratory Standard Institute, 2010). The MICs for tigecycline (MCE, China) were assessed by the broth microdilution method with MH broth (Solaibao, Beijing) in accordance with the Food and Drug Administration breakpoints criteria (FDA, ≤2.0 mg/L is susceptible, 4.0 mg/L is intermediate, and≥8.0 mg/L is resistant) (US FDA, 2013). *Escherichia coli* ATCC 25922 was used as a quality control strain for antimicrobial susceptibility testing. Tigecycline-resistant and carbapenem-resistant *Enterobacteriaceae* (TG-CRE) was defined if *Enterobacteriaceae* was resistant to both at least one carbapenem [imipenem (MIC ≥ 4.0 mg/L) and/or meropenem (MIC ≥ 4.0 mg/L)] and tigecycline (MIC ≥ 8.0 mg/L).

### 2.3 Multilocus sequence typing (MLST) and pulsed-field gel electrophoresis (PFGE)

MLST and PFGE were used to determine the genetic relatedness among the 20 carbapenem-resistant *Klebsiella pneumoniae* (CRKP) and 8 carbapenem-resistant *Enterobacter cloacae* (CRECL) isolates. The analysis focused on seven housekeeping genes from *Klebsiella pneumoniae* (*gapA, infB, mdh, pgi, phoE, rpoB,* and *tonB*) and seven housekeeping genes from *Enterobacter cloacae* (*dnaA, fusA, gyrB, leuS, pyrG, rplB*, and *rpoB*) (Supplementary Table S2). Following polymerase chain reaction (PCR) amplification and Sanger sequencing, the PUBMLST database^2^ was accessed for gene sequence comparison. A single gene sequence number was obtained, which facilitated the determination of the corresponding allele sequence number, ultimately allowing for the identification of the sequence type (ST) of the tested strains (Chavda et al., 2016). For pulsed-field gel electrophoresis (PFGE), small gel blocks were prepared from overnight cultured bacteria. These gel blocks containing bacterial DNA were then cleaved using QuickCut (Rad, USA), and PFGE was conducted on XbaI-digested genomic DNA. The gel was stained for 30 min with Gelred 0.5 mg/mL, and then the fingerprinting profile was observed by the Uvitec system by illuminated UV wave to the gel. *Salmonella enterica* serotype Braenderup strain (H9812) was used as a classical molecular weight standard. The PFGE profiles were compared using In silico stimulation of molecular experiment with a dice similarity coefficient and UPGMA analysis to create the dendrogram. To analyze genetic relatedness, cut-off line at 85% was considered. Data visualization was conducted using BioNumerics version 8.0 software (Park et al., 2020).

### 2.4 Conjugation experiments and bacterial growth curving

To investigate the potential for horizontal transfer of drug-resistance genes to other bacteria, we utilized rifampin-resistant *E. coli* EC600 as the recipient strain, while all samples served as donor strains for conjugation experiments. Single colonies of both donor and recipient bacteria were selected and inoculated into 2 mL of LB broth, followed by incubation on a shaking platform at 37°C for 6 h. Subsequently, 500 μL of both donor and recipient cultures were transferred into 4 mL of LB broth and incubated overnight at 37°C. The cultured bacterial solution was diluted, and 100 μL was applied using an L-shaped inoculating loop onto a double-drug MacConkey plate containing 4 μg/mL tigecycline (Meropenem) and 100 μg/mL rifampicin, followed by incubation for 24 h to select for transconjugants. Concurrently, EC600 was streaked and inoculated on a rifampicin plate as a control. To observe the growth of the transconjugants, they were cultured in 3 mL of LB medium at 200 rpm and 37°C overnight. After adjusting the optical density (OD600) to 0.25, the culture was continued. Every hour, 200 μL was aspirated and replaced with 200 μL of LB medium in a 96-well plate, and the OD600 was measured. This detection process lasted for 24 h, with three duplicate wells for each strain, and C600 served as the control strain (Huang et al., 2023). Growth curves were generated using GraphPad.

### 2.5 Whole genome sequencing and analysis

The DNA in the clinical isolates was extracted by grinding with liquid nitrogen, incubating, cooling at room temperature, centrifuging the supernatant and adding an equal volume of phenol/chloroform/isoamyl alcohol (25:24:1) and mixing with shaking, then centrifuging the supernatant and adding isopropanol and 100 μL of sodium acetate in 2/3 of the volume of the supernatant and mixing, and then precipitating for 2 h at −20°C, centrifuging to remove the supernatant and adding 1 mL of 75% ethanol, and centrifuging again to dissolve the precipitate with TE for second-generation short fragment extraction. After centrifugation again, the precipitate was dissolved with TE for second-generation short fragment extraction, and the magnetic bead method was used to extract the long fragment DNA of the tissue.

The whole genome sequences were sequenced using the Illumina HiSeq platform (San Diego, CA, USA) for all the 31 TG-CRE isolates. *K. pneumoniae* isolate KP28 carrying *tmexC2D2-toprJ2* was subjected to whole-genome sequencing (WGS) using both short-read (Illumina, San Diego, CA, USA) and long-read (PacBio, Menlo Park, CA, USA) platforms. Clean Data was assembled using SPAdes (v3.9.0) assembly software, and the optimal assembly results of each sample were obtained after multiple adjustments. Then, reads will align to the assembled Contig, and the optimization of assemble results would be done through the overlap of the pair end of the reads and Contig. The presence of antimicrobial resistance genes (ARGs) was identified utilizing the CARD (Jia et al., 2017) and ResFinder 4.6.0 server^3^. The NCBI database’s ORFfinder tool^4^ was used to predict the open reading frame present in plasmid 1 of KP28. The BLAST Ring Image Generator (BRIG) v0.95 was employed to generate circular comparisons among the plasmids carrying the *tmexCD-toprJ* genes, with the results visually represented by concentric rings (Alikhan et al., 2011). The EasyFig v2.2.3 software was employed to conduct a comparative analysis of the genetic backgrounds of the strains. All identified *tet*(A) variants protein sequences were aligned with MEGA software, and cluster heatmap of *tet*(A) variants protein was depicted by ChiPlot^5^. The structure-based sequences alignments and 2D structure were performed using ESPript^6^. Phylogenetic trees were generated using kSNP3.0 (Gardner et al., 2015) and then beautified by iTOL v6^7^.

## 3 Results

### 3.1 Clinical and microbiological characteristics of all strains

In 2023, we collected 31 tigecycline-resistant CREs from six hospitals in China, including 16 strains (51.6%) from Suzhou Province, 7 strains (22.6%) from Ningxia Province, 4 strains (12.9%) from Sichuan Province, 2 strains (6.45%) from Beijing Province, 1 strains (3.225%) from Shanghai and 1 strains (3.225%) from Wuhan. Carbapenem phenotyping experiments showed that the number of serinase-producing strains in class A was 13 (42.0%, 13/31), metalloenzyme-producing strains in class B was 9 (29.0%, 9/31), and 9 strains of both enzymes were non-producing (29.0%, 9/31). PCR and Sanger sequencing related results showed that there were 14 strains (45.2%, 14/31) CRE carrying *bla*_KPC_ gene, and all of them were *bla*_KPC-2_ typed, 9 strains (29.0%, 9/31) of CRE carrying *bla*_NDM_ gene, and the typing included 3 strains of *bla*_NDM-1_ gene and 6 strains of *bla*_NDM-5_ gene. One CRE strain each (3.2%, 1/31) carried *bla*_IMP_ and *bla*_OXA-48_ genes. It can be seen that all strains are Multidrug-resistant bacteria (MDR), sensitive to only a few drugs (Table 1). The specific resistance profile was Piperacillin/tazobactam (100%, 31/31), Ceftazidime (100%, 31/31), Cefepime (96.8%, 30/31), Aztreonam (87.1%, 27/31), Imipenem (100%, 31/31), Meropenem (87.1%, 27/31), and Piperacillin/tazobactam (100%, 31/31), Meropenem (87.1%, 27/34), Amikacin (25.8%, 8/31), Ciprofloxacin (74.2%, 23/31), Levofloxacin (77.4%, 24/31) and tigecycline (100%, 31/31).

**Table 1:** Antimicrobial susceptibility testing of TG-CRE, and transconjugants that received the Carbapenem resistance genes of *bla*_KPC_ or *bla*_NDM_.

Strain(s)	MIC (mg/L)^a^									
	TZP	CAZ	FEP	ATM	IPM	MEM	AMK	CIP	LVX	TGC
Clinical isolates										
KP20	≥128R	≥64R	≥32R	≥64R	≥16R	≥16R	≤2S	≥4R	4R	8R
EC25	≥128R	≥64R	≥32R	≥64R	≥16R	1S	16I	≥4R	≥8R	8R
KP27	≥128R	≥64R	≥64R	≥64R	≥16R	≥16R	≤2S	≥4R	≥8R	16R
KP28	≥128R	≥64R	≥64R	≥64R	≤1R	1S	≤2S	≥4R	≥8R	8R
KP31	≥128R	≥64R	≥64R	≥64R	29S	1S	≥64R	≥4R	≥8R	32R
KP32	≥128R	≥64R	≥64R	≥64R	≥16R	≥16R	≥64R	≥4R	≥8R	16R
KA33	≥128R	≥64R	≥64R	≥64R	≥16R	8R	≤2S	≥4R	≥8R	16R
KP34	≥128R	≥64R	≥64R	≥64R	≥16R	≥16R	16S	≥4R	≥8R	16R
KP36	>64R	>32R	>16R	>32R	>8R	>8R	≤8S	>4R	>8R	8R
KP37	>64R	>32R	>16R	>32R	>8R	>8R	>32R	>4R	>8R	16R
KP38	>64R	>32R	>16R	>32R	>8R	>8R	>32R	>4R	>8R	8R
EC39	>64R	>32R	>16R	8S	>8R	>8R	≤8S	>4R	>8R	8R
KP43	≥128R	≥64R	≥64R	≥64R	≥16R	≥16R	≥64R	≥4R	≥8R	8R
KP46	≥128R	≥64R	≥64R	≥64R	≥16R	8R	≤2S	1R	1I	8R
KP47	>64R	>32R	>16R	>32R	>8R	>8R	>32R	>4R	>8R	8R
KP50	>64R	8R	>16R	>32R	>8R	>8R	≤8S	>4R	>8R	8R
KA53	>64R	>32R	16R	>32R	>8R	>8R	≤8S	2I	8R	8R
KP54	>64R	>32R	>16R	>32R	>8R	>8R	≤8S	>4R	>8R	8R
KP57	>64R	>32R	>16R	>32R	>8R	>8R	≤8S	>4R	>8R	16R
KP58	8R	≥64R	2R	≤1S	8R	2I	≤2S	≥4R	4R	16R
KP59	>64R	>32R	>16R	>32R	>8R	>8R	>32R	>4R	>8R	8R
KP60	>64R	>32R	>16R	>32R	>8R	>8R	>32R	>4R	>8R	16R
KA61	>64/R	>32R	4S	>32R	8R	4R	≤8S	≤0.25S	≤0.25S	8R
KP62	>64R	>32R	>16R	>32R	1R	4R	≤8S	>4R	>8R	16R
KP63	64R	≥64R	≥64R	2S	8R	8R	≤2S	≥4R	≥8R	8R
EC67	≥128R	≥64R	≥64R	≤1S	≥16R	≥16R	≤2S	≤0.25S	≤0.25S	8R
EC68	≥128R	18R	≥64R	≥64R	≥16R	11R	≤2S	≤0.25S	1I	8R
EC78	≥128R	≥64R	≥64R	≥64R	>32R	10R	≤2S	≤0.25S	1 I	8R
EC81	≥128R	≥64R	≥64R	≥64R	>32R	10R	≤2S	≤0.25S	1 I	8R
EC89	≥128R	≥64R	≥64R	≥64R	>32R	6R	≤2S	≤0.25S	1I	8R
EC90	8R	≥64R	≥64R	≥64R	>32R	8R	32I	2I	4R	16R
Recipients										
*E. coli* C600	≤4S	≤1S	≤1S	≤1S	≤1S	≤0.25S	≤2S	≤0.25S	0.5S	≤0.125
Transconjugants										
C600/KP46-2^b^	64R	≥64R	8R	≤1S	≥16R	≥16R	≤2S	≤0.25S	0.5S	≤0.125
C600/KP54-1^c^	≥128R	16R	4R	≥64R	≥16R	≥16R	≤2S	≤0.25S	0.5S	≤0.125
C600/KP63^b^	64R	≥64R	≥64R	≤1S	8R	≥16R	≤2S	≤0.25S	0.5S	≤0.125

a TZP, Piperacillin-tazobactam; CAZ, Ceftazidime; FEP, Cefepime; AT-M, Aztreonam; IPM, Imipenem; MEM, Meropenem; AMK, Amikacin; CIP, Ciprofloxac-in; LVX, Levof-loxacin; TGC, Tigecycline.

b *blaNDM-positive transconjugant of E. coli C600, selected by using 100 μg/mL rifampin and 4 μg/mL meropenem.*

c *blaKPC-positive transconjugant of E. coli C600, selected by using 100 μg/mL rifampin an-d 4 μg/mL meropenem.*

### 3.2 Molecular epidemiology based on MLST and PFGE

**Figure 1:**
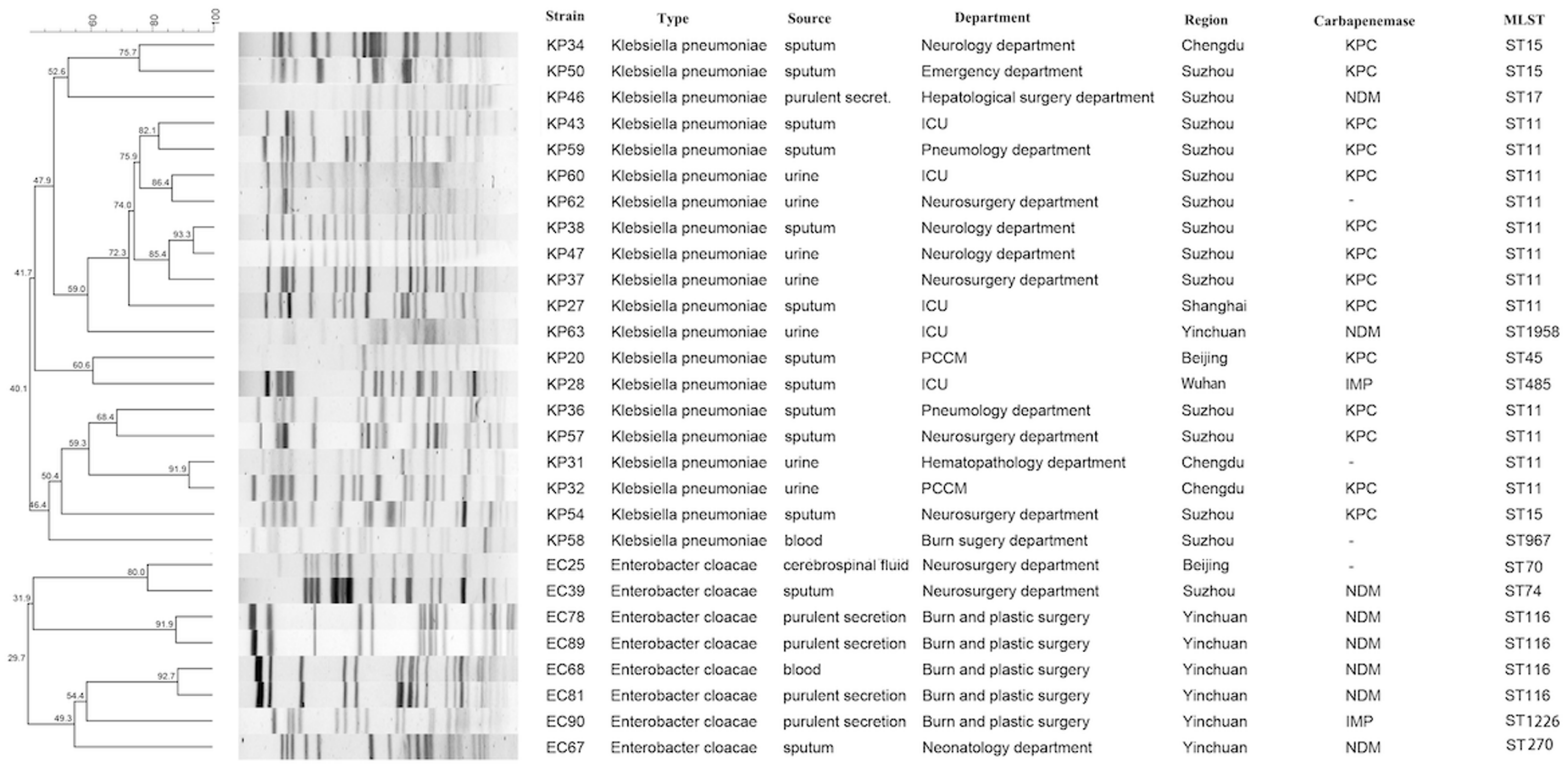
PFGE-based dendrogram of TG-CRE strains. Strain numbers, strain types, source of initial isolation, department, region and carbapenemase are included along each PFGE lane. MLST, multilocus sequence typing; “-”, not detected.

Gel electrophoresis with pulsed fields PFGE classified strains of CRKP, CRECL, and CRKAE into 16 distinct spectra, 6 distinct spectra, and 3 distinct spectra, respectively. Strains from the same region were more likely to have the same spectra; for example, KP38, KP47, and KP37 from Suzhou had the same spectra, and KP31 and KP32 from Chengdu had the same spectra. MLST results revealed that ST11 type dominated CRKP strains with 60% (12/20), followed by ST15 (15%, 3/20), one strain each of ST967, ST1958, ST45, ST485, and ST17, and CRECL strains were dominated by ST116 type (50%, 4/8), followed by ST1226, ST74, ST70, and one strain each of ST270 (Figure 1).

### 3.3 Conjugation assay and fitness cost

**Figure 2:**
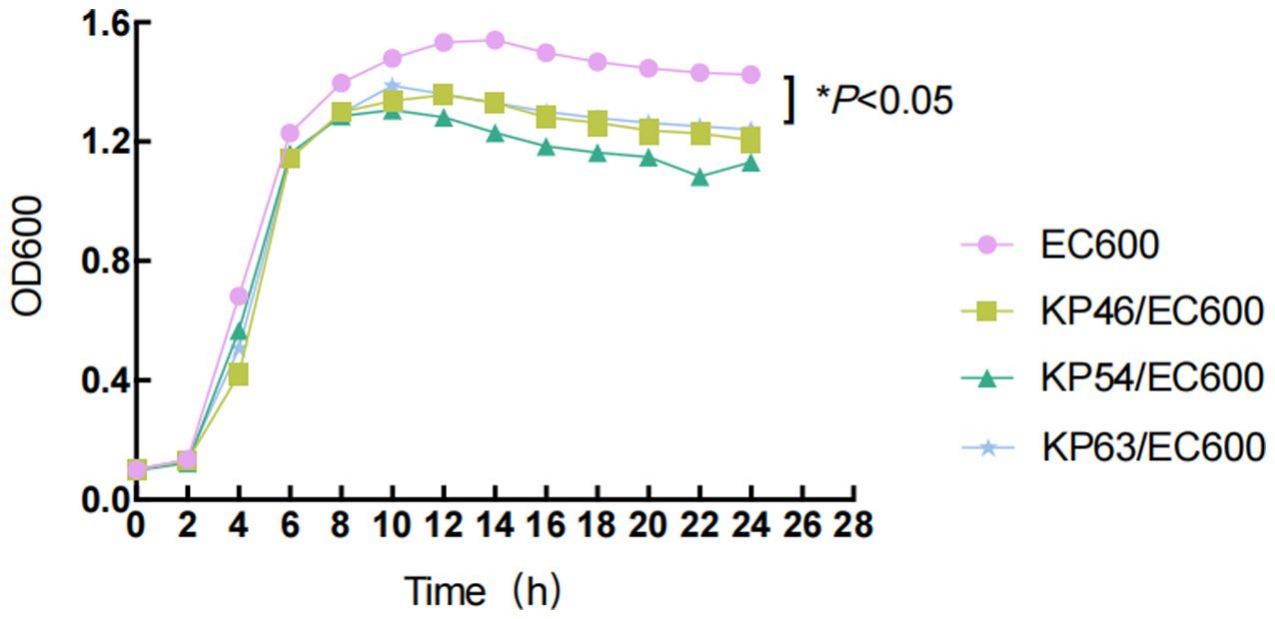
Growth curves of *E. coli* EC600 and the transformants. Values represent the means ± standard deviations obtained from three independent repeated experiments. A repeated measures two-way analysis of variance (ANOVA) with Tukey’s multiple comparisons was used to evaluate statistical significance.

Strains carrying tigecycline resistance genes cannot be transferred to the recipient cell *E. coli* EC600 through plasmid conjugation, however, three strains harboring carbapenem resistance genes, namely KP46, KP54, and KP63, can be successfully transferred. The conjugation frequency was measured at (5.25 ± 2.06) × 10^-6. Consequently, we evaluated the impact of plasmid acquisition on biological adaptability and found a significant difference in the growth rate of *E. coli* EC600 related to plasmid acquisition within the time frame of 4 to 24 h (*p* < 0.05, Figure 2). Stability assays indicated that both the plasmids and their associated resistance genes remained stable in the conjugates after 12 passages without antibiotic selection. Additionally, the antimicrobial susceptibility profiles of translocants containing these plasmids are presented in Table 1.

### 3.4 Identification of antimicrobial resistance genes

**Figure 3:**
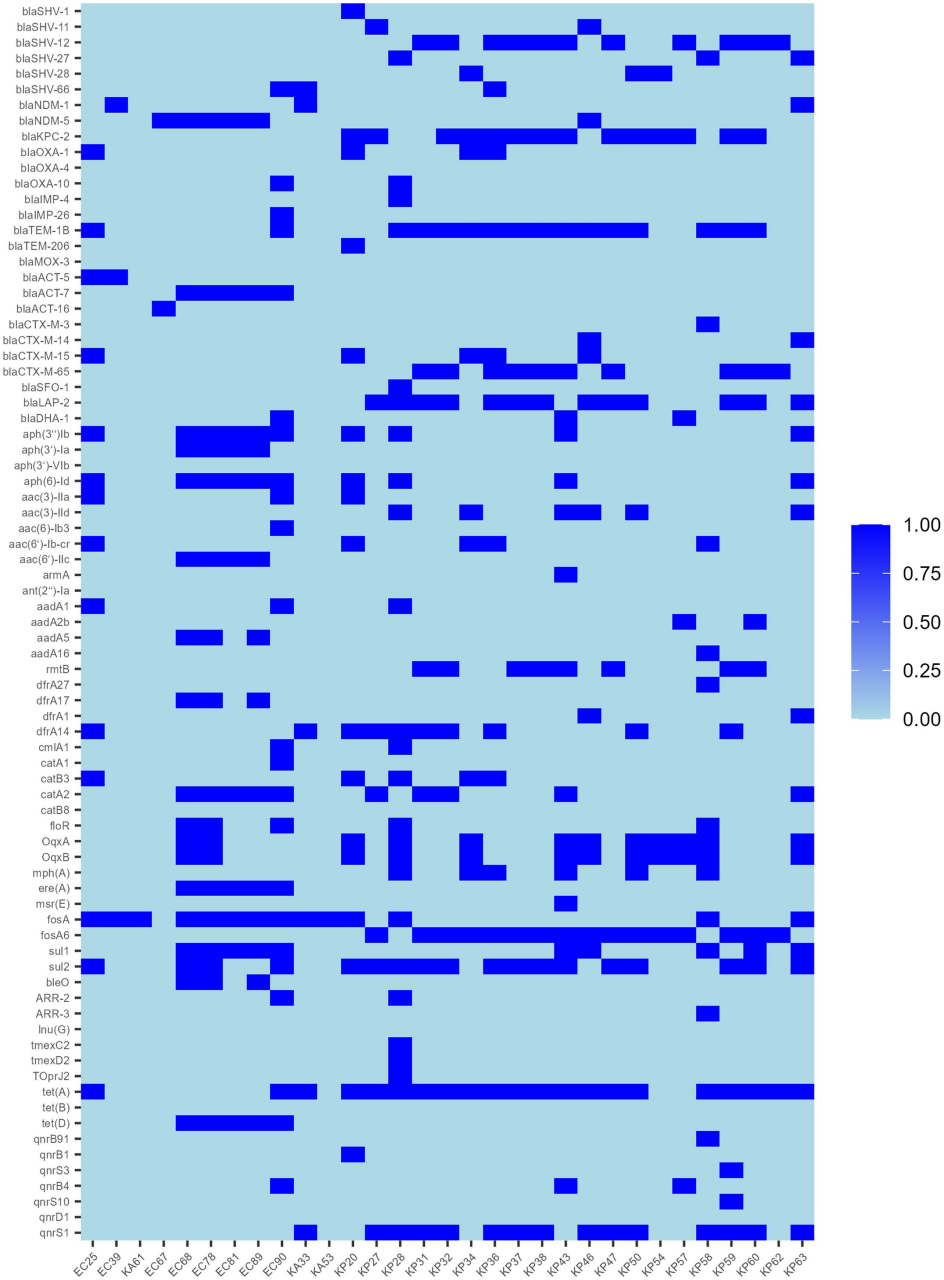
Distribution of antimicrobial resistance genes in 31 TG-CREs.

Resistance genes were predicted from whole genome sequencing data, and the 31 strains contained a total of 79 resistance genes (Figure 3), including carbapenem resistance genes (*bla*_NDM-1_, *bla*_NDM-5_, and *bla*_KPC-2_), ESBL genes, quinolone resistance genes, fluoroquinolone resistance genes, aminoglycoside resistance genes, trimethoprim-like resistance genes, and chloramphenicol resistance genes, macrolide antibiotic resistance genes, tetracycline resistance genes, rifampicin resistance genes and phosphinic acid-sulfonamide-lincomycin resistance genes, and one additional strain, KP28, was found to carry the complete *tmexC2-tmexD2-TOprJ2* tigecycline resistance gene cluster. Sequence comparison of the 21 *tet*(A)-positive strains with the original reference sequence X00006 (GenBank: X00006.1) in the NCBI database revealed that all strain isolates were characterized by non-synonymous mutations in I5R, V55M, I75V, T84A, S201A, F202S, and V203F, and in addition, individual strains had specific mutations, for example, KP62 had I235F, KA33 had A371V, and KP31 had V14L (Supplementary Figure S1).

### 3.5 Genome feature and genetic contexts of the KP28

**Figure 4:**
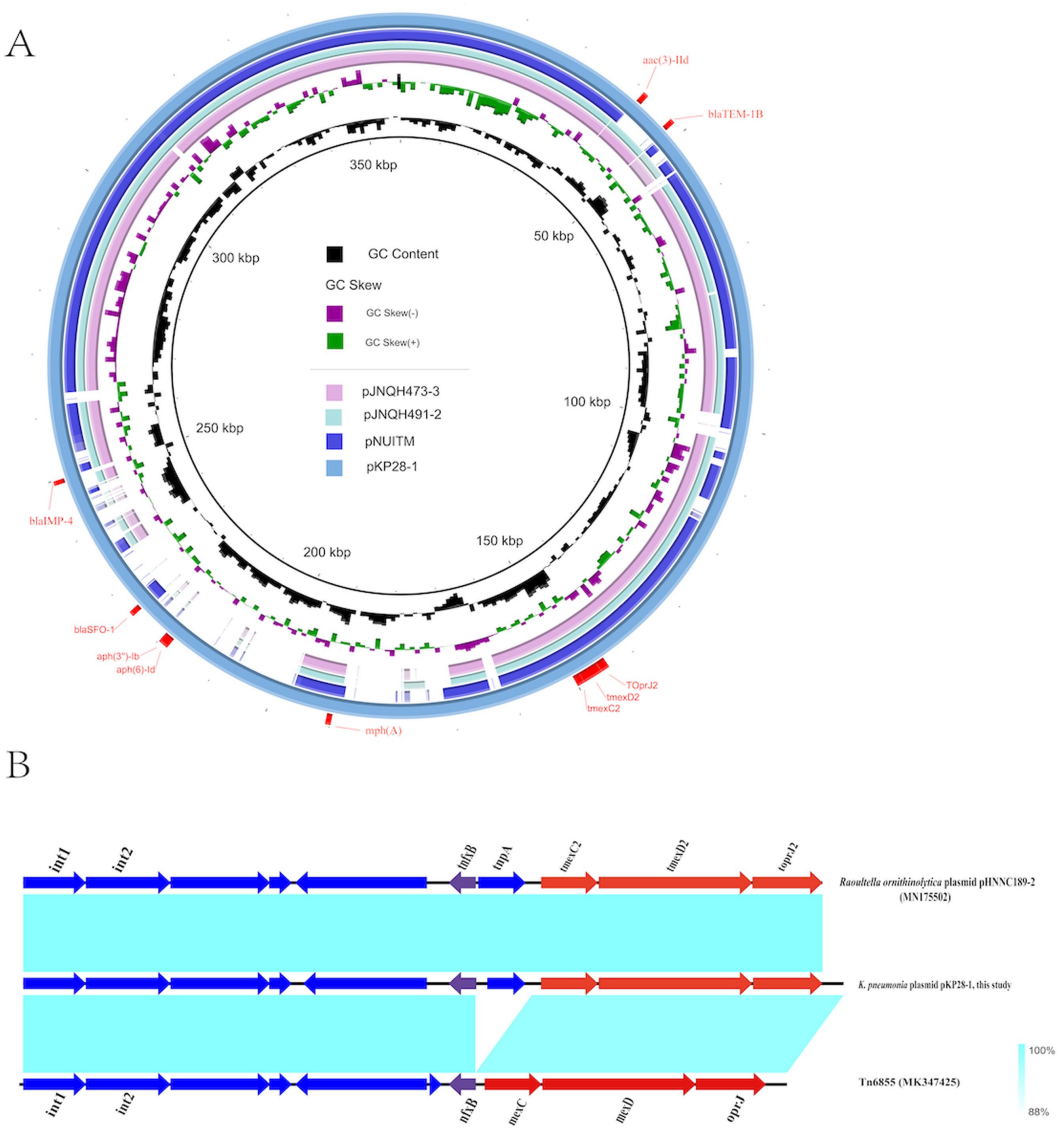
**(A)** Comparative structural analysis of *tmexCD-toprJ*-carrying plasmids pKP28 in this study. **(B)** The genetic context of the *tmexCD2-toprJ2* gene cluster and related mobile elements. pHNNC189-2 (MN175502) and Tn*6855* (MK347425) from the NCBI database were selected as references.

We further sequenced KP28 using the PacBio platform. The genome measured 5,725,141 bp and contained a 5,311,350 bp chromosome as well as two circular plasmids: plasmid pKP28-1 (357,673 bp) and plasmid pKP28-2 (56,118 bp). The IncHI5 plasmid pKP28-1 has 370 predicted open reading frames (ORFs), including a *tmexC2D2-toprJ2* gene cluster, three *β*-lactamase genes (*bla*_IMP-4,_ *bla*_SFO-1_, and *bla*_TEM-1B_), three aminoglycoside resistance genes (*aph(3″)-Ib, aph(6)-Id*, and *aac(3)-IId*), and one macrolide antibiotic resistance gene [*mph(A)*]. BLASTn analysis found that three human IncHI plasmids (pJNQH473-3, pJNQH491-2, and pNUITM) had comparable backbones to pKP28-1 and included the *tmexC2D2-toprJ2* gene (Figure 4A). However, none of the three plasmids contain the *bla*_IMP-4_ gene. There was no conjugative transfer system discovered in pKP28-1, which may explain why they were not successfully transferred into *E. coli* EC600 or *E. coli* J53 in our conjugation experiment.

To comprehensively comprehend the differences in neighboring genetic elements and the genetic environment of *tmexC2D2-toprJ2*, we selected plasmid pHNNC189-2 (GenBank: MN175502) and transposon Tn*6855* (GenBank: MK347425) from the NCBI database as references. Our analysis revealed that the *tnfxB2-tmexCD2-toprJ2* cluster is located within the relevant region of Tn*6855* and highly coincided with the corresponding region of strain *Raoultella ornithinolytica* plasmid pHNNC189-2 (Figure 4B). In contrast to Tn*6855*, the transposon gene tnpA, which encodes transposase, is inserted into the pKP28-1 gene *tnfxB2* in the plasmid of *K. pneumoniae*. Tn*6855* and its variants are predominantly found in *Klebsiella pneumoniae* plasmids and *Raoultella ornithinolytica* plasmids, however, the fundamental mechanism underlying the spread of Tn*6855* remains unclear. Further research is warranted.

### 3.6 Possible origin of *tnfxB*2-*tmexC2D2-toprJ2*

**Figure 5:**
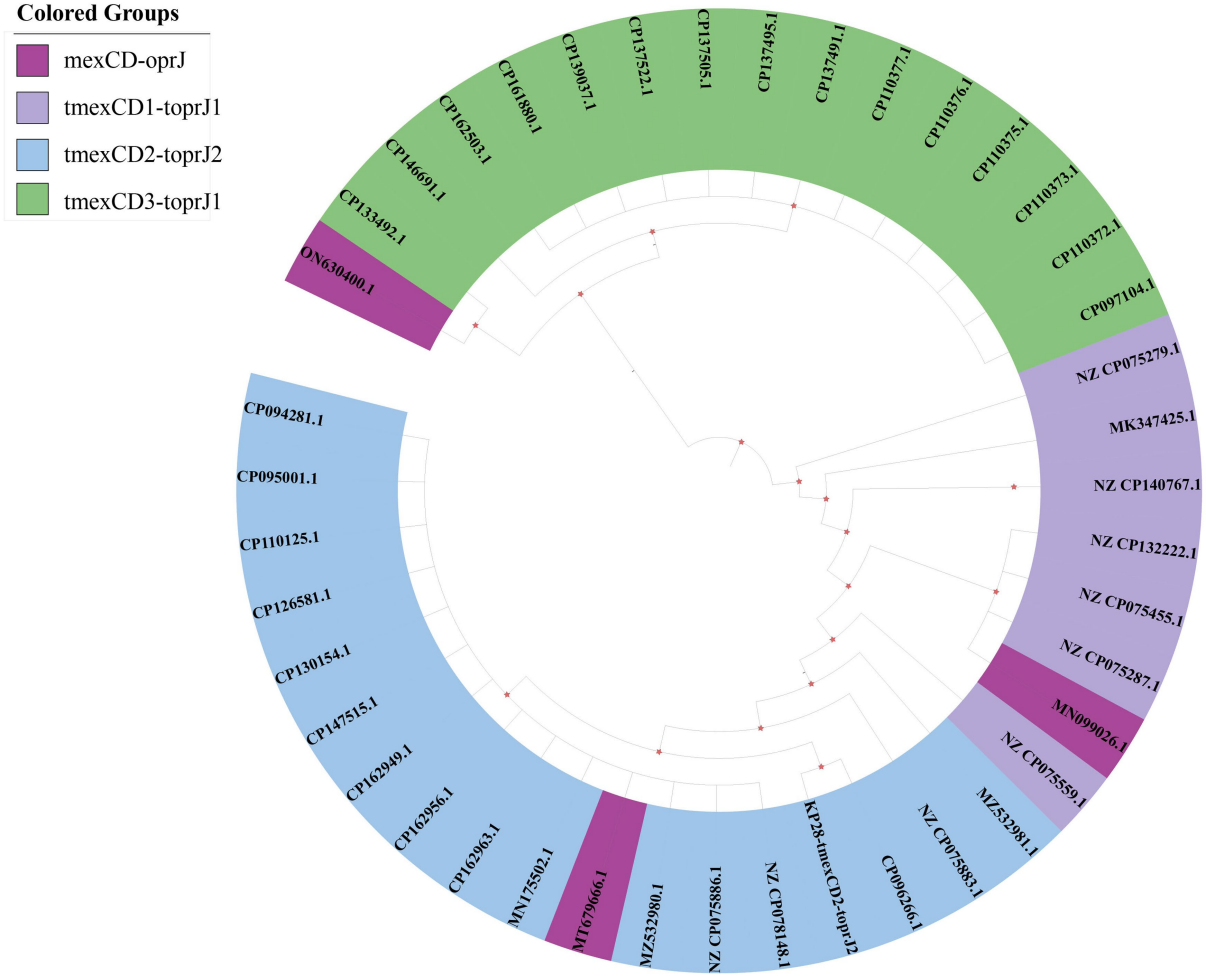
Phylogenetic tree of the *tmexCD-toprJ* gene clusters. The different background colors of the sequence IDs indicate the related variants of *mexCD-oprJ*.

To elucidate the origin and evolutionary relationship of the *tnfxB2-tmexCD2-toprJ2* RND drug efflux complex, we conducted a search of the NCBI genome database for strains containing the *mexCD-oprJ* and *tmexCD-toprJ* gene clusters. This search revealed that 113 strains contained the *mexCD-oprJ* gene cluster, while 8,120 strains contained the *tmexCD-toprJ* gene cluster. Applying the criteria that each strain’s uploaded sequence exceeds 200 kb and fully covers the *mexCD-oprJ* or *tmexCD-toprJ* gene, we screened 43 strains. The resulting neighbor-joining tree places *tmexC2D2-toprJ2* within a phylogenetic group characterized by high amino acid identity (>90%) (Figure 5). Notably, the *tmexCD2-toprJ2* gene is predominantly located in *Klebsiella* and *Raoultella* species. The phylogenetic tree indicates that the variant KP28-*tmexCD2-toprJ2* identified in this study is highly similar to CP096266.1, which was isolated from Zhengzhou, China, in 2022, as recorded in the NCBI database. This similarity suggests a potential risk of transfer and transmission between the two variants (Figure 5). Additionally, most *mexCD-oprJ* and *tmexCD-toprJ* variants were found in plasmids, highlighting the role of plasmids as significant intermediaries that carry gene clusters facilitating transmission between bacteria. Previous studies have established that *mexCD-oprJ* variants generally emerge earlier than *tmexCD-toprJ* variant (Alikhan et al., 2011). Our findings suggest that *tmexCD2-toprJ2* will likely evolve from *tmexCD1-toprJ1*, while *tmexCD3-toprJ* is distantly related.

## 4 Discussion

Over the past two decades, carbapenem-resistant *Enterobacteriaceae* (CRE) have emerged as a major challenge in hospital infections globally (Giacobbe et al., 2024). These bacteria gain resistance primarily by acquiring carbapenemase genes, which enable them to break down carbapenems, a class of antibiotics (Alizadeh et al., 2020). Plasmid-mediated carbapenemases are a key factor in this resistance development, allowing the rapid spread of resistance genes among different bacterial species (van Duin and Doi, 2017). Tigecycline, a last-resort antibiotic for treating extensively drug-resistant bacteria, is facing threats from emerging resistance mechanisms (He et al., 2019). In 2019, He et al. identified plasmid-mediated high-level mobility of tigecycline resistance genes *tet*(X3) and *tet*(X4). Additionally, there have been cases where *tet*(X) coexists with other resistance genes like *tmexCD3-toprJ3* and *bla*_NDM-4_ in *Enterobacteriaceae* (Hirabayashi et al., 2021). This highlights the limited treatment options available when patients become infected with this type of bacteria.

In this study, we conducted a comprehensive microbiological characterization of 31 tigecycline-resistant, carbapenem-resistant *Enterobacteriaceae* (TG-CRE) strains collected from multiple hospitals in China. Our findings indicate that all the strains are multidrug-resistant (MDR), with some even exhibiting extensive drug resistance. It is particularly noteworthy that strains carrying tigecycline resistance genes cannot be transferred to recipient cells via plasmid conjugation, whereas strains harboring carbapenem resistance genes can be successfully transferred, with a relatively high frequency of conjugation. This observation suggests that tigecycline resistance genes may be transmitted through other mechanisms, such as chromosomal mutations or plasmid integration, while carbapenemresistance genes are more likely to be disseminated through conjugation. Furthermore, strains that acquire resistance plasmids exhibit significant differences in biological adaptability, which may influence their survival and propagation in the natural environment. The MLST typing in this study revealed that *Klebsiella pneumoniae* was dominated by ST11 (60%), followed by ST15. The results were consistent with the overall epidemic situation in China. *Enterobacter cloacae* was mainly composed of ST116 (50%), which was different from the previously reported ST418 type in China (Jin et al., 2018). PFGE further confirmed that the strains from the same region (such as KP37, KP38, and KP47 from Suzhou) had high homology, suggesting a high degree of clonal transmission within the hospital or region. The isolates from different cities were less related to each other. In this work, conjugation techniques were used to successfully transfer the *bla*_KPC_ and *bla*_NDM_ genes from the donor *Klebsiella pneumoniae* to *E. coli* EC600. The persistence of the plasmids and the conjugants’ resistance to carbapenems suggested that CRE might disseminate the resistance by plasmid-level transmission, despite the conjugation efficiency being relatively low. Notably, the conjugants carrying plasmids showed a loss in growth adaptability, which is in line with the “cost of adaptation” theory put forward in earlier research. Through compensating changes, the resistant plasmids may restore their adaptive advantage, but their long-term durability under natural settings may be limited. We must be more vigilant in order to stop the spread.

This study identified 79 resistance genes through whole-genome sequencing and drug resistance gene prediction, including known carbapenem resistance genes such as *bla*_KPC-2_, *bla*_NDM-1,_ and *bla*_NDM-5_, as well as a variety of other categories of drug resistance genes, which aligns with previous drug susceptibility results. Literature indicates that mutations in the tetracycline resistance gene *tet*(A) can confer resistance to tigecycline (Chiu et al., 2017). In this study, 21 strains were identified based on the reference sequence of the *E. coli* plasmid RP1 tetracycline resistance determinant (GenBank:X00006). All TG-CRE isolates carrying the *tet*(A) gene exhibited non-synonymous mutations, specifically 5R, V55M, I75V, T84A, S201A, F202S, and V203F, including double frame-shifting mutations (S201A, F202S, V203F). This finding is consistent with the analysis by Zhang et al. (2023), suggesting that these mutations may bea primary factor contributing to the resistance observed in these strains. Particular attention was given to the IncHI5-like hybrid plasmid found in one of the low-level tigecycline-resistant CRKP isolates, which co-carried *tmexC2D2-toprJ2* and *bla*_IMP-4_ but was unable to undergo successful conjugative transfer. The presence of the transcriptional repressor *tnfxB*2 may have contributed to this low level of horizontal resistance. Linear comparison analysis revealed that *tnfxB2-tmexC2D2-toprJ2* is situated within a specific region of transposon Tn*6855.* However, it is noteworthy that the *tnpA* gene is inserted between *tnfxB2* and *tmexC*2. The *tnpA* gene, a common genetic marker found in certain transposons (Tn), encodes a transposase. This enzyme can specifically recognize the DNA sequences at both ends of the receptor target and Tn, facilitating the integration of Tn into the target sequence (Karvelis et al., 2021). Tn*6855* is a prototype integrative and mobilizable element (IME) that includes attL/R (attachment sites at the left and right ends), two genes encoding putative site-specific integrases (int1 and int2), four hypothetical protein genes, and the *nfxB-mexCD-oprJ* cluster, as previously described (Yu et al., 2021). It is hypothesized that the Tn*6855* variant identified in this study likely originates from a plasmid of *Raoultella ornithinolytica*. However, the specific transmission mechanism has yet to be investigated. In 2019, large IncFIB (Mar)/IncHI1B and IncHI5-like hybrid plasmids, along with the IncFrepB (R1701) plasmid co-carrying carbapenemase-encoding genes (*bla*_IMP-4,_ *bla*_NDM-1_, and *bla*_KPC-2_) and *tmexCD-toprJ*, were reported. However, unlike the IncFrepB (R1701) plasmid, these large plasmids exhibited no conjugation effect on either *E. coli* or *K. pneumoniae* (Guo et al., 2024), which accounts for the failure of conjugation. Nevertheless, the evolution of plasmids presents potential clinical risks that warrant our close attention.

In our study, the sequences collected from the NCBI database revealed that the bacterial genera harboring the *tmexCD1-toprJ1* and *tmexCD2-toprJ2* gene clusters were predominantly *Klebsiella*, while the bacterial genera containing the *tmexCD3-toprJ1* gene cluster were primarily Pseudomonas. Notably, most of the *tmexCD-toprJ* gene clusters are located on plasmids, suggesting a significant risk of interspecies transfer upon entering *Enterobacteriaceae*. The *tmexCD2-toprJ2* gene cluster may have originated from the chromosome of Pseudomonas (Wang et al., 2021) and exhibits a high nucleotide similarity to *tmexCD1-toprJ1*. However, it possesses a weaker promoter, *tmexC2*, and a repressor factor, *tnfxB*2 (Wang et al., 2024), which correlates with lower resistance levels. *Klebsiella pneumoniae* associated with the *tmexCD2-toprJ2* gene cluster is globally distributed across various environments and hosts, with the majority of isolates originating from China. This indicates that China plays a crucial role in the dissemination of the global *tmexCD2-toprJ2* gene cluster (Yao et al., 2024). Our sample size is limited, preventing us from making specific statistical inferences about the positivity rate in the region. We have only conducted preliminary explorations of the resistance mechanisms of all strains. Additionally, unreported variants of *tet*(A) necessitate further construction of vectors to verify their resistance.

## 5 Conclusion

Our study is part of a multi-center investigation and has revealed the emergence of carbapenem-resistant *Enterobacteriaceae* that are resistant to tigecycline, a last-resort antibiotic, in hospitals across multiple regions. This finding was confirmed through whole-genome sequencing analysis. Most strains examined carry mutants of the *tet*(A) gene, which substantiates the mechanism behind tigecycline resistance. Notably, only one CRKP strain was found to carry the *tmexCD-toprJ* gene cluster, suggesting that its overall incidence remains relatively low. However, strains harboring the *bla*_KPC_ and *bla*_NDM_ carbapenem resistance genes have the potential for horizontal transmission. To date, clonal transmission of the *tmexCD2-toprJ2* and the *bla*_IMP-4_ co-carried plasmid IncHI5 has not been observed. Given the increasing use of tigecycline in the treatment of clinical CRE infections, ongoing surveillance is essential.

## Data Availability

The original contributions presented in the study are included in the article/Supplementary material, further inquiries can be directed to the corresponding authors.

## Footnotes

^1^ https://blast.ncbi.nlm.nih.gov/blast.cgi

^2^ https://pubmlst.org/bigsdb?db=pubmlst_mlst_seqdef&page=batchSequenceQuery

^3^ http://genepi.food.dtu.dk/resfinder

^4^ https://www.ncbi.nlm.nih.gov/orffinder/

^5^ https://www.chiplot.online/

^6^ http://espript.ibcp.fr/ESPript/cgi-bin/ESPript.cgi

^7^ https://itol.embl.de/
