# Supplementary material for: Epidemiology and resistance mechanisms of tigecycline- and carbapenem-resistant Enterobacteriaceae in China: a multicentre genome-based study
Source: Front Microbiol. 2025 May 7;16:1582851. doi: 10.3389/fmicb.2025.1582851 (PMC12092438; doi:10.3389/fmicb.2025.1582851)
Supplement: Supplementary file 1 [file Data_Sheet_1.docx]

Supplementary Material

**Supplementary information**

**Table S1 The primers for Carbapenem resistance genes**

| **Target gene** | **Primer sequence（5’→ 3’）** | **Annealing temperature℃** | **Product length（bp）** | |
| --- | --- | --- | --- | --- |
| *bla*_KPC_ | F:ATGTCACTGTATCGCCGTCT | 56 | | 893 |
|  | R:TTTTCAGAGCCTTACTGCCC |  |  |  |
| *bla*_NDM_ | F:ATGGAATTGCCCAATATTATGC | 56 | | 813 |
|  | R: TCAGCGCAGCTTGTCGG |  |  |  |
| *bla*_IMP_ | F: TGAGCAAGTTATCTGTATTC  R: TTAGTTGCTTGGTTTTGATG | 55 | | 740 |
| *bla*_OXA-48_ | F: GCGTGGTTAAGGATGAACAC | 57 | | 438 |
|  | R: CATCAAGTTCAACCCAACCG |  |  |  |

**Table S2 The primers for house-keeping genes**

|  | **Target gene** | **Primer sequence（5’→ 3’）** | **Annealing temperature℃** |
| --- | --- | --- | --- |
| *klebsiella pneumoniae* | *rpoB* | F:GGCGAAATGGCWGAGAACCA  R:GAGTCTTCGAAGTTGTAACC | 53 |
|  | *gapA* | F:TGAAATATGACTCCACTCACGG  R:CTTCAGAAGCGGCTTTGATGGCTT | 63 |
|  | *mdh* | F:CCCAACTCGCTTCAGGTTCAG | 53 |
|  |  | R:CCGTTTTTCCCCAGCAGCAG |  |
|  | *pgi* | F:GAGAAAAACCTGCCTGTACTGCTGGC | 53 |
|  |  | R:CGCGCCACGCTTTATAGCGGTTAAT |  |
|  | *phoE* | F:ACCTACCGCAACACCGACTTCTTCGG  R:TGATCAGAACTGGTAGGTGAT | 53 |
|  | *infB* | F:CTCGCTGCTGGACTATATTCG  R:CGCTTTCAGCTCAAGAACTTC | 53 |
|  | *tonB* | F:CTTTATACCTCGGTACATCAGGTT | 48 |
|  |  | R:ATTCGCCGGCTGRGCRGAGAG |  |
| *enterobacter cloacae* | *dnaA* | F:AYAACCCGCTGTTCCTBTATGGCGGCAC | 50 |
|  |  | R:KGCCAGCGCCATCGCCATCTGACGCGG |  |
|  | *fusA* | F:TCGCGTTCGTTAACAAAATGGACCGTAT | 50 |
|  |  | R:TCGCCAGACGGCCCAGAGCCAGACCCAT |  |
|  | *gyrB* | F:TCGACGAAGCGCTCGCGGGTCACTGTAA | 50 |
|  |  | R:GCAGAACCGCCCGCGGAGTCCCCTTCCA |  |
|  | *leuS* | F:GATCARCTSCCGGTKATCCTGCCGGAAG | 50 |
|  |  | R:ATAGCCGCAATTGCGGTATTGAAGGTCT |  |
|  | *pyrG* | F:AYCCBGAYGTBATTGCRCAYMAGGCGAT | 50 |
|  |  | R:GCRCGRATYTCVCCCTSHTCGTCCCAGC |  |
|  | *rplB* | F:GTAAACCGACATCTCCGGGTCGTCGCCA | 50 |
|  |  | R:ACCTTTGGTCTGAACGCCCCACGGAGTT |  |
|  | *rpoB* | F:AAAAACGTATTCGTAAGGATTTTGGTAA | 50 |
|  |  | R:CCAGCAGATCCAGGCTCAGCTCCATGTT |  |


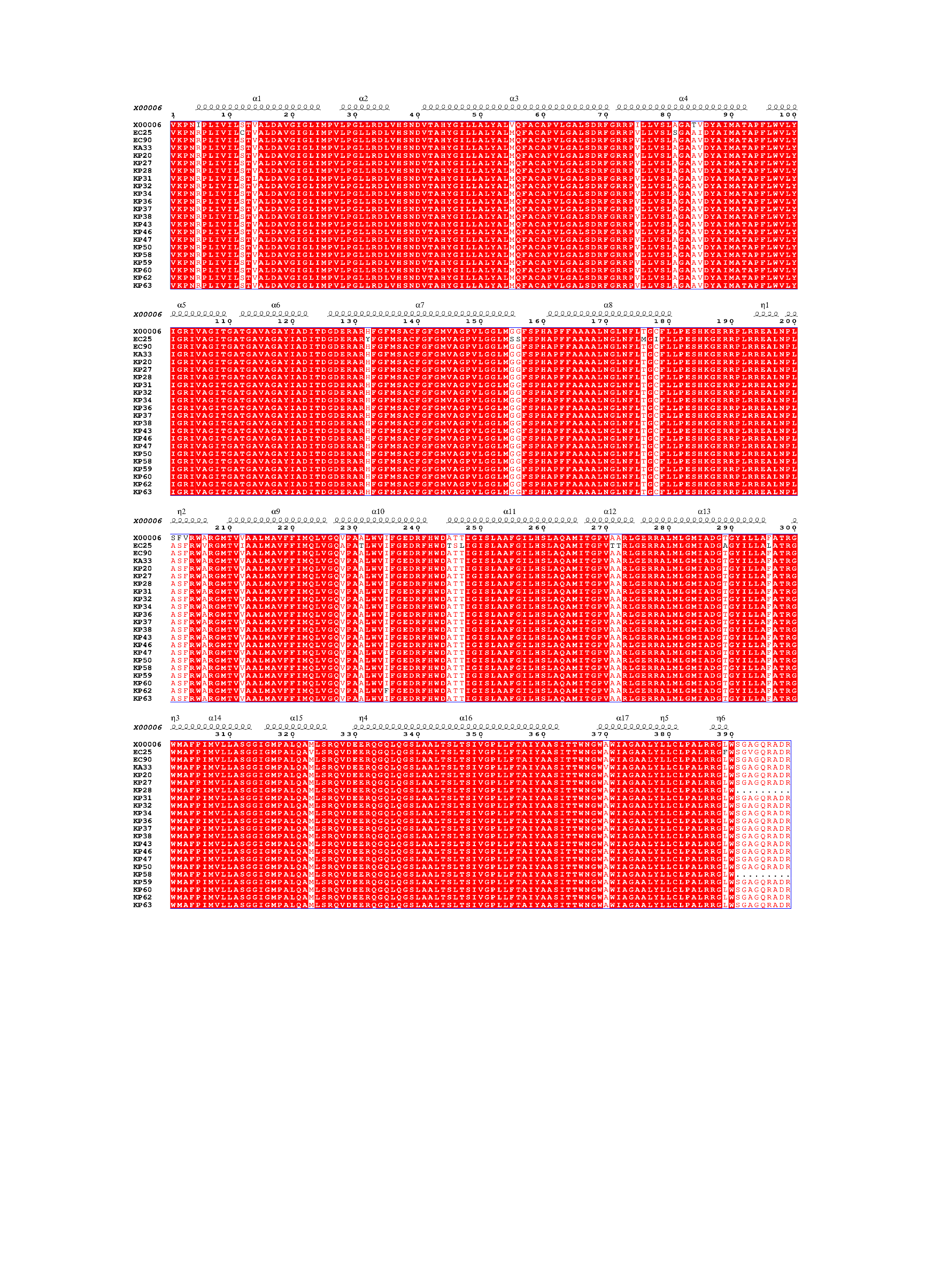


**Supplementary Figure S1:** Amino acid differences of 21 *tet*(A) variants in our study
